# Stress Granules and Neurodegenerative Disorders: A Scoping Review

**DOI:** 10.3389/fnagi.2021.650740

**Published:** 2021-06-23

**Authors:** Mohammad Reza Asadi, Marziyeh Sadat Moslehian, Hani Sabaie, Abbas Jalaiei, Soudeh Ghafouri-Fard, Mohammad Taheri, Maryam Rezazadeh

**Affiliations:** ^1^Molecular Medicine Research Center, Tabriz University of Medical Sciences, Tabriz, Iran; ^2^Department of Medical Genetics, Faculty of Medicine, Tabriz University of Medical Sciences, Tabriz, Iran; ^3^Department of Medical Genetics, School of Medicine, Shahid Beheshti University of Medical Sciences, Tehran, Iran; ^4^Skull Base Research Center, Loghman Hakim Hospital, Shahid Beheshti University of Medical Sciences, Tehran, Iran

**Keywords:** stress granules, pathological aggregations, neurodegenerative disorders, amyotrophic lateral sclerosis, Alzheimer's, TDP-43, TIA-1, PABP-1

## Abstract

Cytoplasmic ribonucleoproteins called stress granules (SGs) are considered as one of the main cellular solutions against stress. Their temporary presence ends with stress relief. Any factor such as chronic stress or mutations in the structure of the components of SGs that lead to their permanent presence can affect their interactions with pathological aggregations and increase the degenerative effects. SGs involved in RNA mechanisms are important factors in the pathophysiology of neurodegenerative disorders such as amyotrophic lateral sclerosis (ALS), frontotemporal degeneration (FTD), and Alzheimer's diseases (AD). Although many studies have been performed in the field of SGs and neurodegenerative disorders, so far, no systematic studies have been executed in this field. The purpose of this study is to provide a comprehensive perspective of all studies about the role of SGs in the pathogenesis of neurodegenerative disorders with a focus on the protein ingredients of these granules. This scoping review is based on a six-stage methodology structure and the PRISMA guideline. A systematic search of seven databases for qualified articles was conducted until December 2020. Publications were screened independently by two reviewers and quantitative and qualitative analysis was performed on the extracted data. Bioinformatics analysis was used to plot the network and predict interprotein interactions. In addition, GO analysis was performed. A total of 48 articles were identified that comply the inclusion criteria. Most studies on neurodegenerative diseases have been conducted on ALS, AD, and FTD using human post mortem tissues. Human derived cell line studies have been used only in ALS. A total 29 genes of protein components of SGs have been studied, the most important of which are TDP-43, TIA-1, PABP-1. Bioinformatics studies have predicted 15 proteins to interact with the protein components of SGs, which may be the constituents of SGs. Understanding the interactions between SGs and pathological aggregations in neurodegenerative diseases can provide new targets for treatment of these disorders.

## Introduction

Cell function is divided between the organelles sited inside the cell. Based on the presence of lipid membrane, organelles can be divided into main two groups. Nucleus, mitochondria, endoplasmic reticulum, and Golgi apparatus are the major membranous organelles. Membraneless organelles are ribosomes (Turi et al., 2019), stress granules (Arrigo et al., 1988), p-body (Sheth and Parker, 2003), and nucleolus (Shaw and Jordan, 1995) that are formed during a process called the liquid-liquid phase separation phase (Marnik and Updike, 2019). The existence of stress at the cellular level can lead to a variety of responses, including global translational inhibition, leading to the formation of stress granules (SG) (Aulas et al., 2018). SGs, membraneless ribonucleoproteins containing mRNA, are cytoplasmic accumulations being stopped at the initiation of translation and disappear after the end of stress induction (Boncella et al., 2020). These stresses in mammalian cells include viral infections (biotic stress), induction of redox stress with sodium arsenite, heat and UV radiation which are environmental stress conditions (Kedersha et al., 2013). Three groups can form the protein component of stress granules: RNA-binding proteins, translation initiation factors, and non-RNA-binding proteins (Cao et al., 2020; Samadian et al., 2021). Stoppage at the critical stage of translation initiation due to biotic or environmental stress leads to the isolation of the translating polysomes resulting in the creation of a huge reservoir of RNA and related proteins that build and increase the number of SGs. On the other hand, relieving stress and increasing translated mRNAs is associated with disassembly and reduction in the number of these granules (Panas et al., 2016; Marnik and Updike, 2019).

There are two mechanisms for stopping translation initiation at the cellular level. Phosphorylation of the α subunit of eIF2 transcription initiation factor and prevention of the eIF-4F complex assembly. eIF2 is present in the ternary complex and is responsible for transferring the initiator tRNA to the pre-initiation complex at the 5'-ends of mRNAs. The result of eIF2 phosphorylation is reduction in its binding to GTP and loss of its ability to transfer the initiator tRNA to ribosomes for start codon recognition. Four stress associated kinases (HRI, PERK, PKR, GCN2) have the ability to phosphorylate the α subunit in the eIF2 factor (Aulas et al., 2017; Wolozin and Ivanov, 2019). eIF-4F complex is responsible for detecting the structure of the cap at the 5' mRNA end, and assembly of this complex is controlled by the PI3K-mTOR kinase cascade. eIF4E is inactivated in phosphorylated form and this phosphorylation is performed by mTOR, a member of the phosphatidylinositol 3-kinase-related kinase family of protein kinases (Mitra et al., 2015). eIF4E in active mode prevents the eIF-4F complex assembly and the translation process halts at the initiation point (Gingras et al., 1999; Aulas et al., 2017; Wolozin and Ivanov, 2019).

No specific function can be considered for SGs, but they can be considered as a “decision point” (Wolozin and Ivanov, 2019) for the fate of mRNA trapped in its structure. SG can define the fate of an mRNA that could be subjected to storage, degeneration, or re-initiation of translation. The probability of mRNA being in the SGs structure is sequence-independent but is directly related to the low translatability and the increase in the length of encoded region as well as the UTR region in the mRNA (Khong et al., 2017). Since proteins and RNAs with important roles can be included in SGs, this would affect biological interactions (Arimoto-Matsuzaki et al., 2008).

Traces of SGs have been found in many diseases, such as cancer (Gao et al., 2019), neurodegenerative disorders (Wolozin, 2012) and autoimmune conditions (McCormick and Khaperskyy, 2017). Neurodegenerative diseases include as amyotrophic lateral sclerosis (ALS) (Baron et al., 2013), Alzheimer's disease (AD) (Ash et al., 2014), and multiple sclerosis (MS) (Salapa et al., 2018). The neurodegeneration process involves atrophy and loss of neuronal activity (Wolozin and Ivanov, 2019). In neurodegenerative diseases, many mutations and misfolding events have been identified in this protein components of SGs, which can lead to the accumulation of abnormal proteins and the formation of SGs. Pathological symptoms appear when the presence of SGs becomes permanent due to an increase in their formation resulting from chronic stress (Brown et al., 2020) and a decrease in their deletion due to mutations in genes involved in the process of autophagy (Chitiprolu et al., 2018; Brown et al., 2020).

So far, many studies have been done on the nature of SGs, their components, structures and their pathological characteristics in various neurodegenerative diseases, and useful results have been obtained. In this study, we tried to establish a strong correlation between clinical evidence and genetic characteristics in neurodegenerative diseases in the form of a scoping review study by summarizing all human clinical studies and human-derived cell lines in the field of SGs.

## Methods

### General Framework for Review

The writing strategy of this article is based on the methodology proposed by Arksey and O'Malley (2005). This strategy was later improved by Levac et al. (2010) and Colquhoun et al. (2014). In this review, 5 steps of the 6-step framework have been followed which includes (1) Development of research questions, (2) Search strategy, (3) Study eligibility criteria, (4) Data extraction, (5) Collating, summarizing and reporting the results. The sixth step, consultation, is optional and is not included in this article. Also, in order to observe the principles of clarity and transparency in writing the article, the Preferred Reporting Items for Systematic Reviews and Meta-Analyses Extension for Scoping Reviews (PRISMA-ScR) Checklist has been used well (Tricco et al., 2018).

### Development of Research Questions

To survey, summarize and discuss the studies on SGs in human and human-derived cell lines in neurodegenerative diseases, our review was guided by the following questions:

1. What exactly have been done on SGs in humans with neurodegenerative disease?
2. Exactly what studies have been done on SGs in human-derived cell lines in neurodegenerative disease?

### Search Strategy

The publications were searched using seven databases: PubMed, Scopus, Cochrane, Google Scholar, Embase, Web of Science, and ProQuest, based on the search method of each database and was not limited to the date, language, subject or type of publication. Also, review articles published in this field were reviewed to reduce the possibility of losing related articles. “Neurodegeneration” keyword was the medical subject heading (MeSH) used in search strategy if available. PubMed and Embase related search strategies are shown in Appendix I. The last search was conducted on December 8, 2020. The references were managed using EndNote X8.1.

### Study Eligibility Criteria

Studies in neurodegenerative diseases in relation to SGs in humans or in human-derived cell lines were screened from publications obtained during the search process. All types of publications were reviewed, including journal articles, conference presentations, conference abstracts and reports. Non-English articles with English abstracts were also included. Screening was done in two stages. At first, both researchers (MRA, MSM) screened articles separately based on title and abstract, according to the inclusion criteria mentioned above. In the next step, full texts of the selected articles were investigated to measure its relevance to the research question. Finally, appropriate articles were selected based on the eligibility criteria. Any contradiction was resolved in agreement with the opinion of the third person.

### Data Extraction

Two separate charts were designed for human samples and human-derived cell lines in Microsoft Excel to help extracting the data. Chart related to human sample articles included author's name, year of publication, diagnosed neurodegenerative disease (number of patients), age and country, sample and method of analysis, protein component of SGs and major findings. The chart related to human-derived cell lines articles included the author's name, year of publication, origin of cell lines, age at biopsy, country, sample, method of analysis, mutation, and major findings. Two researchers (MRA, MSM) separately extracted data from articles based on charts.

### Collating, Summarizing, and Reporting the Results

Quantitative and qualitative analysis was performed on the data obtained from the publications. In the quantitative analysis section, a descriptive numerical summary of the extent, nature and distribution of the studies were reviewed. In the qualitative analysis section, based on the research question mentioned earlier, a narrative review was performed on the available information with affirmation on the broader context suggested by Levac et al. (2010).

### Bioinformatics Analysis

Two disease-protein interaction networks were designed using Cytoscape v3.8.0 software (Shannon et al., 2003) based on data extracted from articles. One was the network of neurodegenerative diseases with protein components of SGs and their interactions according to the results of human studies and the other was the protein components of SGs studied in human-derived cell lines from ALS. Therefore, the gene/proteins found from the literature search were used as input. The output was the interaction network between these proteins. Gene ontology (GO) analysis was performed using Enrichr's web-based tools and services (Kuleshov et al., 2016) on the genes of SGs protein components in neurodegenerative diseases and ALS. Protein-protein interaction was predicted using the string-db cytoscape plugin (Doncheva et al., 2019) on the data extracted from the articles. The analyzed graph of GO was ranked based on *p* < 0.05.

## Result

**Figure 1:**
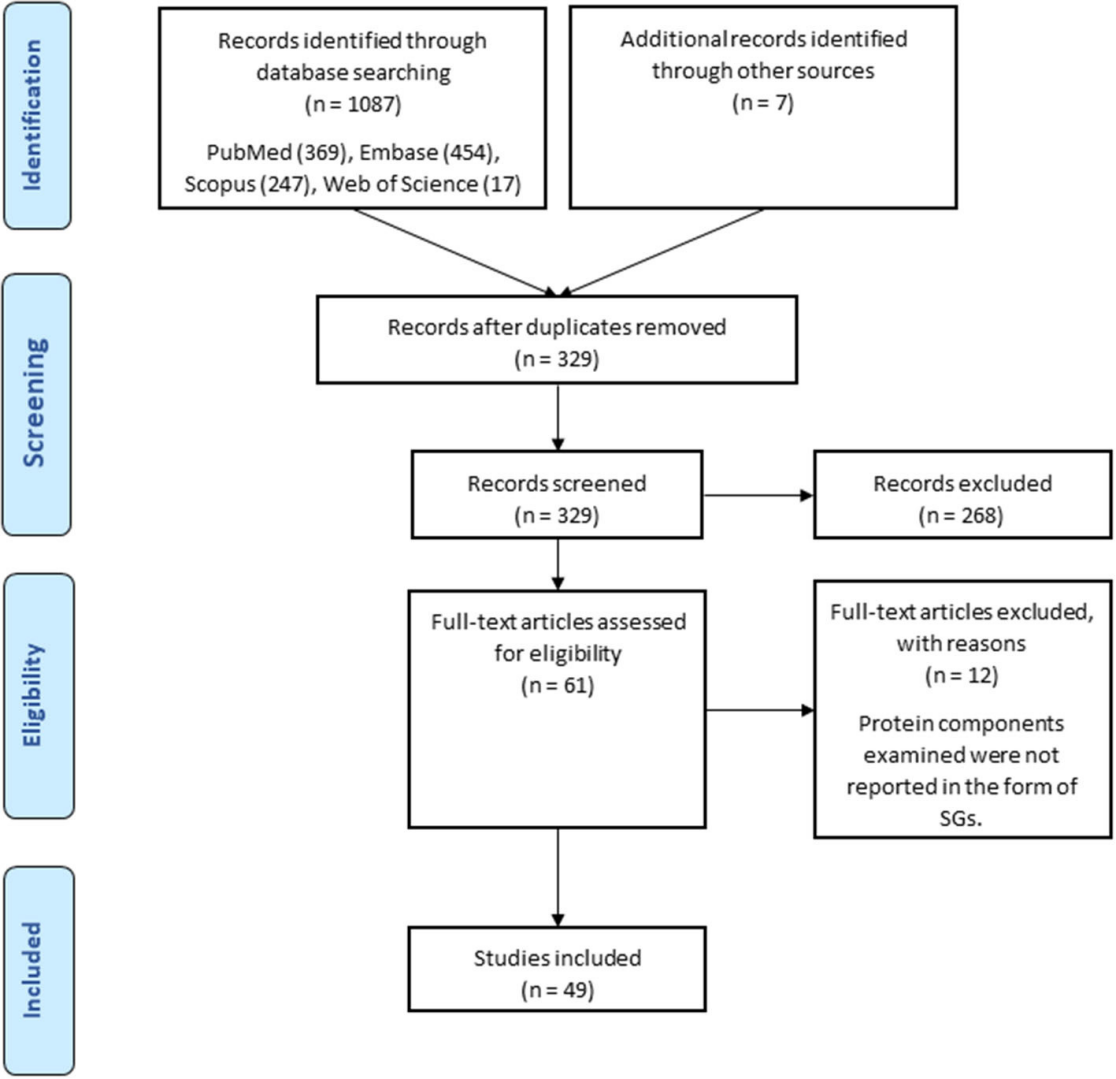
Search strategy flow chart based on the PRISMA flow diagram.

Keyword-based searches in various databases yielded 1,087 related records. Also, 7 records from other sources were added to the results. Of these, 758 duplicates were identified. After excluding the duplicates, the number of articles with potential to be related to the research question reached 329. A total of 262 articles were excluded from the study by screening titles and abstracts and 61 articles remained. By reviewing the full text of the remaining articles, 49 articles were included in the study, of which 5 articles were conference abstracts. The process of selecting eligible studies is detailed in the flowchart in Figure 1. Table 1 provides an overview of the included articles.

**Table 1:** Detailed characteristics of included publications.

**Characteristic**	**Number (*n =* 49)**	**Percentage (%)**
Type		
Journal article	44	89.7
Conference abstract	5	10.20
Year		
<2010	3	6.12
2010–2015	8	16.32
2015–2020	38	77.55

### Summary of Methods of Studies Performed in Human Subjects

**Figure 2:**
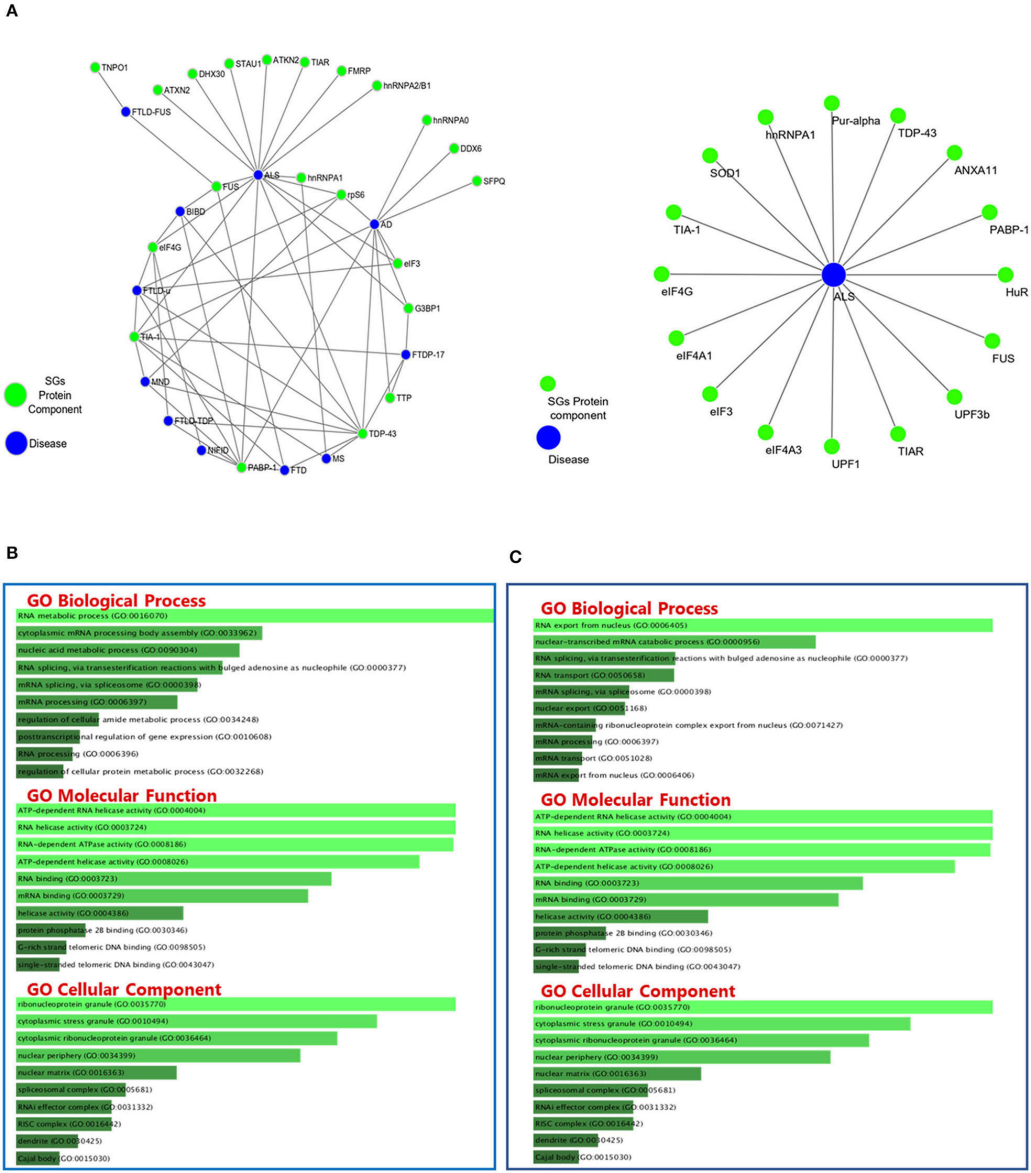
Protein-Protein Interactions and Top 10 GO analysis of target genes in SGs protein components in neurodegenerative disease. The network of neurodegenerative diseases with protein components of SGs and their interactions. **(A)** Gene ontology analysis of the genes in Table 2 **(B)** and Table 3 **(C)** has been performed. The length of each bar represents the degree of significance in that particular category sorted by *p-*value. Note that the lower the color intensity of the bars, the greater the relationship with that category.

The studies were published from 2008 to 2020. The total number of neurodegenerative cases in this study is 258. These studies were conducted ALS (Fujita et al., 2008; Colombrita et al., 2009; Volkening et al., 2009; Dormann et al., 2010; Liu-Yesucevitz et al., 2010; Bentmann et al., 2012; Farg et al., 2013, 2014; McGurk et al., 2014; Cohen et al., 2015; Lim et al., 2016; Manghera et al., 2016; Dreser et al., 2017; Hirsch-Reinshagen et al., 2017; Mackenzie et al., 2017; Bennett et al., 2018; Chen and Cohen, 2019; Mann et al., 2019; Montalbano et al., 2020; Vassileff et al., 2020), ALS/FTD (Hirsch-Reinshagen et al., 2017; Mackenzie et al., 2017), ALS/frontotemporal lobar degeneration (FTLD) (Mann et al., 2019), AD (Castellani et al., 2011; Vanderweyde et al., 2012; Ivanov et al., 2016; Maziuk et al., 2018; Silva et al., 2019; Montalbano et al., 2020; Younas et al., 2020), MS (Salapa et al., 2018, 2020; Levin et al., 2020), FTD (McGurk et al., 2014; Montalbano et al., 2020), FTDP-17 (Vanderweyde et al., 2012), FTLD-u (Fujita et al., 2008; Dormann et al., 2010; Liu-Yesucevitz et al., 2010), FTLD with TDP-43 inclusions (FTLD-TDP) (Dormann et al., 2010; Bentmann et al., 2012; Cohen et al., 2015), FTLD-FUS (Hock et al., 2018), neuronal intermediate filament inclusion disease (NIFID) (Dormann et al., 2010), basophilic inclusion body disease (BIBD) (Dormann et al., 2010; McGurk et al., 2014) and motor neuron disease (MND) (Fujita et al., 2008), which are summarized in Table 2. Cases in this study include both sexes, but because the gender of the cases was not mentioned in many studies, it was not indexed in Table 2. The samples used in the studies were brain, spinal cord and blood, of which twelve studies used only brain (Castellani et al., 2011; Vanderweyde et al., 2012; Manghera et al., 2016; Hock et al., 2018; Maziuk et al., 2018; Salapa et al., 2018, 2020; Silva et al., 2019; Levin et al., 2020; Montalbano et al., 2020; Vassileff et al., 2020; Younas et al., 2020), seven studies used only spinal cord (Colombrita et al., 2009; Farg et al., 2013, 2014; McGurk et al., 2014; Dreser et al., 2017; Mackenzie et al., 2017; Chen and Cohen, 2019), ten studies used both tissues (Fujita et al., 2008; Volkening et al., 2009; Dormann et al., 2010; Liu-Yesucevitz et al., 2010; Bentmann et al., 2012; Cohen et al., 2015; Lim et al., 2016; Hirsch-Reinshagen et al., 2017; Bennett et al., 2018; Mann et al., 2019) and one study used blood samples (Ivanov et al., 2016). The analysis methods of most studies were immunohistochemistry (IHC), but immunoprecipitation (IP) (Volkening et al., 2009; Farg et al., 2013; Montalbano et al., 2020), immunofluorescent microscope imager (Ivanov et al., 2016), RNA-FISH (Mann et al., 2019), mass spectrometry (Vassileff et al., 2020) and confocal microscopy (Volkening et al., 2009; Montalbano et al., 2020) were also used. The protein component of the SGs examined in the studies is also summarized in Figure 2. The included studies have been conducted in the United States, Canada, Australia, Germany, the Netherlands, Japan, Italy, Russia, South Korea, China and the United Kingdom. Based on the bioinformatics studies using the string-db Cytoscape plugin, 15 new genes were predicted to interact with the genes extracted from these studies, including HNRNPC, HNRNPDL, KHDRBS1, HNRNPH1, EDC4, DCP1A, ELAVL1, MATR3, TAF15, HNRNPF, PTBP1, CAPRIN1, PABPC1, KHSRP, AND ILF3.

**Table 2:** Stress Granules and neurodegenerative disorders in human samples.

**Author(s)**	**Year of publication**	**Country**	**Diagnosed neurodegenerative disease (number of patients)**	**Age**	**Sample**	**Analysis method(s)**	**Protein component of stress granules**	**Major findings**	**References**
Fujita et al.	2008	Japan	•MND (2) •ALS (3) •FTLD-u (2)	MND Patients (45 and 58 years old) ALS patients (63.7 and 81 years old) FTLD-u with MND patients (62 and 63 years old)	Spinal cord Brain (hippocampus)	IHC	PABP-1 TIA-1 TDP-43 rpS6	•The presence of RNA in the Basophilic inclusions in the diagnosed disorders. •The Basophilic inclusions were labeled for PABP-1, TIA-1 and ribosomal protein S6; In contrast the BIs were not immune-positive for TDP-43.	Fujita et al., 2008
Volkening et al.	2009	Canada	•ALS (7(3 SOD associated)	NR	Spinal cord Brain	IP and western blot Biotin-labeled NFL 3′UTR RNA, IHC and confocal microscopy, RNA-IP-PCR	TDP-43 TIA-1 STAUFEN SOD1	•TDP-43 showed colocalization in ALS motor neurons with mutants or WT-SOD1. •TDP-43 showed colocalization in ALS's MNs and controls with STAUFEN. •The frequency of SGs and P-bodies in ALS's MNs were higher than controls and showed colocalization with TDP-43. •NFL mRNA processing in ALS's MNs is severely altered by TDP-43.	Volkening et al., 2009
Colombrita et al.	2009	Italy	•ALS (3)	NR	Spinal cord	IHC	TDP-43 TIAR HuR	•Mis-localization of TDP-43 was evident in the cytoplasm as a granular distribution. •No co-localization was observed between TIAR and HuR with TDP-43 inclusions.	Colombrita et al., 2009
Liu-Yesucevitz et al.	2010	United States	•ALS (4) •FTLD-u (5)	ALS Patients ranging from 63 to 79, mean 69 years FTLD-U Patients ranging from 72 to 83, mean 75.6 years	Spinal cord Brain (Frontal Cortex)	IHC	TDP-43 eIF3 TIA-1	•Colocalization were found between pTDP-43 inclusions and TIA-1 and eIF3 proteins. •Inclusions containing TDP-43 in ALS's spinal cord and FTD's brain can contain stress granule proteins.	Liu-Yesucevitz et al., 2010
Dormann et al.	2010	Canada	•ALS (1) •FTLD-u (3) •NIFID (3) •BIBD (1) •FTLD-TDP (2)	NR	Spinal cord Brain (hippocampus)	IHC	PABP-1 eIF4G	•PABP-1 and eIF4G are present in NCIs in patients with FUS pathology. •PABP-1 IHC shows highly immunoreactivity in NCIs in ALS's spinal cord MNs, BIBD, FTLD-u's hippocampus and NIFID but not detected in FTLD-TDP's hippocampus. •PABP-1 colocalized with p62 in same brain regions in ALS, FTLD-u, NIFID and BIBD but not in FTLD-TDP. •eIF4G was not detected in FTLD-TDP.	Dormann et al., 2010
Castellani et al.	2011	United States	AD (13)	Patients ranging from 67 to 89, mean 77.7 years	Brain (hippocampus)	IHC	rpS6	•IHC analysis showed that neurons in the AD's hippocampus containing 20 times more rPS6-positive granules compared to age-matched groups. •rPS6-positive granules were more common in neurons lacking NFT. •GVD granules colocalized with rPS6 in pyramidal neurons.	Castellani et al., 2011
Bentmann et al.	2012	Germany	•FTLD-TDP (5) •ALS (4)	NR	Brain (hippocampus) Spinal cord	IHC	TDP-43 PABP-1	•In ALS's spinal cord TDP-43 (N-terminal and C-terminal) labeled in NCIs but in FTLD-TDP's hippocampus only C-terminal of TDP-43 were labeled. •Double-labeling of PABP-1 and pTDP-43 of the same cases showed positive inclusions in spinal cord but not in cortical inclusions.	Bentmann et al., 2012
								•Inclusions in the FTLD's cortex, which have C-terminal fragments, were not labeled with PABP-1, whereas components in the spinal cord, which include full-length TDP-43, were positive for this protein marker.	
Vanderweyde et al.	2012	United States	•AD (6) •FTDP-17 (5)	AD's Patients ranging from 65 to 96, mean 82.5 years FTDP-17 Patients ranging from 44 to 53, mean 50.6 years	Brain (hippocampus) Brain (Frontal Cortex)	IHC	TIA-1 TDP-43 TTP G3BP1	•In ADs, TIA-1 colocalization was observed with Tau aggregations (pathological-phosphorylated-total) in all cases. •Larger Tau aggregations tended to be more colocalized with TIA-1 than smaller one. •Examination of controls showed small sparse aggregations of Tau. Also, by examining older controls, the presence of SGs in them was confirmed but they were not related to Tau pathology and its aggregations. •FUS inclusions moderately colocalized with TIA-1 inclusions, TDP-43 colocalized with TIA-1 inclusions infrequently. •Presence of TTP and G3BP1 in AD's brain was confirmed. G3BP1 not aggregated with TIA-1. TTP strongly colocalized with Tau inclusions. •Similar results observed in 5 cases of FTDP-17. •TIA-1-positive SGs were observed in both microglia, and astrocytes cell types.	Vanderweyde et al., 2012
Farg et al.	2012	Australia	ALS (3)	ALS Patients ranging from 42 to 55, mean 50 years	Spinal cord	IHC IP	ATXN2 FUS	•Ataxin-2 is present in the structure of SGs, which rapidly respond to stress and insults that affect the cell, and preventing translation of incorporate mRNA. •In controls, ataxin-2 was rarely observed in FUS inclusions Whereas in the ALS's, ataxin-2 was often seen with FUS cytoplasmic inclusions. •IP revealed that FUS was co-precipitated with ataxin-2 and not in controls. •Interaction between ataxin-2 and FUS is also RNA-independent, which was completed with RNase treatment.	Farg et al., 2013
McGurk et al.	2014	United States	•ALS [14 (2 with C9orf72 mutation and 3 with ATXN2 mutation)] •ALS-D [8 (6 with C9orf72 mutation and 1 with ATXN2 mutation)] •FTD (1) •BIBD (2)	ALS Patients ranging from 52 to 79, mean 65.7 years ALS-D Patients ranging from 46 to 67, mean 55.8 years FTD Patient with 47 years old BIBD patients with 65 and 75 years old	Spinal cord	IHC	PABP-1 TDP-43 FUS	•PABP-1 is colocalized with mature TDP-43 inclusions, not with TDP-43 pre-inclusions. •PABP-1, colocalized with TDP-43 inclusions, in the ALS's with ATXN2 and C9orf72 mutations. •The frequency of PABP-1 colocalization with TDP-43 inclusions in ALS's without mutation is 36%, in ALS's with ATXN2 mutation is 47% and in ALS's with C9orf72 mutation is 67%. •In patients with FUS pathology, PABP-1 observed with pathologic FUS in the motor neurons.	McGurk et al., 2014
Farg et al.	2014	Australia	ALS (1)	74 years old patient	Spinal cord	IHC	hnRNPA1 hnRNPA2B1	•Increased colocalization between C9orf72 and rab7 and rab11 in ALS's compared to controls •Poor regulation of cell trafficking in patients with C9orf72 mutation	Farg et al., 2014
Cohen et al.	2015	United States	•ALS (6) •FTLD-TDP (3)	ALS Patients ranging from 39 to 81, mean 56.8 years FTLD Patients ranging from 54 to 75, mean 64 years	Spinal cord Brain	IHC	TDP-43	•FTLD brain was highly immunoreactive for TDP-43 inclusions. •TDP-43 is full length (N-/C-terminal) and highly acetylated in ALS's TDP-43 inclusions. •Changes in acetylation plays an important role in loss of function and accumulation of TDP-43 in stress granules.	Cohen et al., 2015
Manghera et al.	2016	United States	ALS (5)	ALS Patients ranging from 50 to 76, mean 61.4 years	Brain (frontal cortex)	IHC	TDP-43 G3BP1	•Simultaneous expression of ERVK and TDP-43 is one of the hallmarks of ALS. •An increase in expression of G3BP1 was seen in ALSs vs. controls. This may be due to the increased expression of TDP-43 seen in ERVK+ cortical neurons. •No colocalization was observed between G3BP1 and ERVK. •SGs and viroplasms segregation can be a trigger to increased expression of ERVK viral proteins in ALS.	Manghera et al., 2016
Ivanov et al.	2016	Russia	AD(26)	AD's Patients ranging from 72 to 82, mean 76 years	Blood	MI	eIF3	•Significant heterogeneity in the distribution of eIF3 in neutrophils of patients' blood samples compared to controls. •Heterogeneity of eIF3 in patients' neutrophils is associated with the formation of SGs.	Ivanov et al., 2016
Lim et al.	2016	•South Korea	ALS (2)	ALS Patients with 34 and 57 years old, mean 45.5 years	Brain Spinal cord	IHC	FUS	•Distribution of FUS in controls and ALS samples is limited to the nucleus. •Oxidative stress induces the amassment of cytoplasmic FUS in stress granules, thus mimicking pathological characteristics recognized in mutant FUS in ALS patient. •Cytoplasmic aggregation of FUS was observed in G504Wfs mutant carrier sample in contrast to the control and ALS samples. •In G504Wfs mutant ALSs, FUS proteins emerges from the nucleus and undergoes cytoplasmic localization.	Lim et al., 2016
Dreser et al.	2017	Netherland	ALS (28(9 with c9orf72 mutation and 4 with FUS pathology)	NR	Spinal cord	IHC	MATRIN-3	•Cytoplasmic accumulations of matrin-3 were detected in C9orf72 and FUS cases. •Increased nuclear matrin-3 staining in all cases.	Dreser et al., 2017
Hirsch-Reinshagen et al.	2017	Canada	•ALS (1) •ALS/FTD (4)	ALS Patients with FTD ranging from 30 to 79, mean 58 years ALS Patient with 59 years old	Spinal cord Brain	IHC	TDP-43 TIA-1 PABP-1	•IHC showed a large number of granular TDP-43 in prefrontal cortex and primary motor cortex. •In all cases, Hippocampal dentate granule cells and dopaminergic neurons in the substantia nigra was affected. •NCIs were seen in granular, filamentous, round and compact form in LMN. •IHC failed to show abnormality using a number of TIA-1 antibodies. •The staining patterns were similar in ALSs with and without TIA-1 mutation and with normal control group, and no aggregations were detected in cases with TIA-1 mutation. •Colocalization between TIA-1 and pTDP-43 was not detected.	Hirsch-Reinshagen et al., 2017
Mackenzie et al.	2017	United States	ALS/FTD (5)	ALS Patients with FTD ranging from 30 to 79, mean 58.2 years	Spinal cord	IHC	TDP-43 TIA-1	•Numerous rounds and hyaline TDP-43 inclusions were observed in all five autopsy cases. •TIA-1 mutations increase the tendency of TIA-1 protein to phase transition.	Mackenzie et al., 2017
Bennett et al.	2018	•United States	ALS (2)	NR	Brain Spinal cord	IHC	TDP-43	•TDP-43 was accumulated in stress granules seen in the cytoplasm of ALS lumbar cord MNs. •These cytosolic accumulations were associated with nuclear clearance. •TDP-43 ectopic position is a feature of ALS in humans.	Bennett et al., 2018
Silva et al.	2018	•United States	AD (NR)	NR	Brain (Temporal cortex)	IHC	DDX6 PABP-1	•IHC revealed DDX6 and PABP-1 were localized around p-TAUs. •The proximity and increased presence of DDX6 and PABP-1 can be attributed to the pathology caused by Tau aggregations.	Silva et al., 2019
Hock et al.	2018	•United kingdom	FTLD-FUS (4)	NR	Brain	IHC	FUS TNPO1	•Hypertonic stress leads to cytoplasmic transmission and loss of neuronal FUS function in SGs-independent manner. •Hypertonic Stress-mediated FUS transmission is caused by disruption of nuclear imports mediated by transport (TNPO1). •FTLD's frontal cortex was positive for TNPO1 neuronal cytoplasmic inclusion. •Astrocytes are resistant to FUS transmission and damage caused by hypertonic stress.	Hock et al., 2018
Maziuk et al.	2018	•United States	AD (7)	AD's Patients ranging from 57 to 97, mean 81.4 years	Brain	IHC	DDX6 hnRNPA0	•DDX6 and hnRNPA0 IHC indicated the presence of RBP inclusions in the cortex. •Location of RBPs indicated an inverse association compared to mature NFTs. It was observed that RBPs are found as pathological inclusions around mature tangles, thus RBP aggregates form near pathological TAUs but are strongly separated from them.	Maziuk et al., 2018
Salapa et al.	2018	•Canada	MS (1)	51 years old patient	Brain	IHC	TIA-1 hnRNPA1	•TIA-1 and hnRNPA1 normally have nuclear and cytoplasmic distribution in contrast, In MS's hnRNPA1 nuclear depletion was observed in brain neurons. •Accumulations of TIA-1 in large SGs formed in the cytoplasm of MS's brain neurons, but not in the nucleus. •In MS's brain neurons unlike control, hnRNPA1 and TIA-1 colocalized in cytoplasmic granules in double-label staining.	Salapa et al., 2018
Chen et al.	2019	•United States	ALS (6)	ALS Patients ranging from 39 to 81, mean 56.8 years.	Spinal cord	IHC	FMRP TDP-43 TIA-1 TIAR	•The observed immunohistochemical patterns of FMRP and p-FMRP are cytoplasmic and not related to the pathology of TDP-43. •Different forms of TDP-43 with FMRP and p-FMRP co-localized in Double-Labeling. •TIA-1 and TIAR Double-labeling presented limited colocalization with TDP-43 inclusions. •TIA-1 was placed next to the TDP-43 inclusions and TIAR showed a stippled pattern in the cytoplasm of MNs and were not directly colocalized with TDP-43 inclusions.	Chen and Cohen, 2019
Mann et al.	2019	United States	ALS/FTLD (NR)	NR	Brain (hippocampus) Spinal cord	IHC RNA-FISH	TDP-43 G3BP1 ATKN2	•optoTDP-43 accumulations are similar to TDP-43 accumulations in TDP-43 proteinopathy. •RNA-FISH performed on ALSs' spinal cord and FTLDs' brain with a poly-T probe and failed to found TDP-43/mRNA colocalization. TDP-43 inclusions lacked mRNA. •No colocalization between TDP-43 and G3BP1/ATKN2 inclusions was detected.	Mann et al., 2019
Salapa et al.	2020	•Canada	MS (12)	MS Patients ranging from 44 to 65, mean 52.1 years.	Brain	IHC	TDP-43 hnRNPA1	Localization of TDP-43 and hnRNPA1 in MS's showed severe nuclear depletion and robust cytoplasmic localization compared with controls.	Salapa et al., 2020
Younas et al.	2020	•Germany	AD (16)	AD's Patients ranging from 56 to 93, mean 77.7 years	Brain	IHC	SFPQ TIA-1	•Examination of SFPQ in AD's brain tissue showed down-regulation. •SFPQ in AD's brain showed nuclear depletion and cytoplasmic colocalization with TIA-1. •SFPQ showed extra-nuclear colocalization with p-Tau in AD's brain legions. •There is probably a link between SFPQ and Tau oligomers in oligomerization and misfolding.	Younas et al., 2020
Vassileff et al.	2020	•Australia	ALS (10)	NR	Brain	Mass Spectrometry	STAU1 DHX30 TDP-43	•MECV examination in ALS compared to controls identified 16 protein packages that were statistically significant. •Up-regulation of two basic proteins from RNA-binding proteins in the dynamic pathway of SGs in ALS compared to controls.	Vassileff et al., 2020
Levin et al.	2020	•Canada	MS (14)	NR	Brain	IHC	TDP-43 hnRNPA1	•Nuclear depletion and cytoplasmic localization of TDP 43 in MS's neurons compared with controls. •Both TDP-43 and hnRNPA1 were colocalized in structure of SGs in the cytoplasm.	Levin et al., 2020
Montalbano et al.	2020	•United States	•AD (3) •ALS (3) •FTD (2)	AD's Patients ranging from 81 to 86, mean 83.3 years. ALS Patients ranging from 64 to 84, mean 72 years. FTD Patients with 56 and 60 years old, mean 58 years.	Brain (frontal cortex)	IHC IP confocal microscopy	TDP-43	•The interaction between the Tau and TDP-43 may be involved in the pathogenesis of AD, ALS and FTD. •TDP-43 is detected in stress granules in FTD and ALS.	Montalbano et al., 2020

*SG, Stress Granule; MND, motor neuron disease; ALS, Amyotrophic lateral sclerosis; FTLD-u, Frontotemporal lobar degeneration; IHC, Immunohistochemistry; BI, Basophilic inclusion; IP, immunoprecipitation; PCR, polymerase chain reaction; WT, Wild Type; MN, Motor Neurons; FTD, Frontotemporal Degeneration; NIFID, Neuronal intermediate filament inclusion disease; BIBD, Basophilic Inclusion Body Disease; NCI, neuronal cytoplasmic inclusion; AD, Alzheimer's disease; NFT, Neurofibrillary tangle; GVD, granule vascular degeneration; ALS-D, Amyotrophic lateral sclerosis with dementia; ERVK, Endogenous retrovirus-K; LMN, Lower Motor Neurons; RBP, RNA Binding Protein; MS, Multiple Sclerosis; MECV, Motor cortex extracellular vesicles*.

### Summary of Methods of Studies Performed in Human-Derived Cell Lines

**Figure 3:**
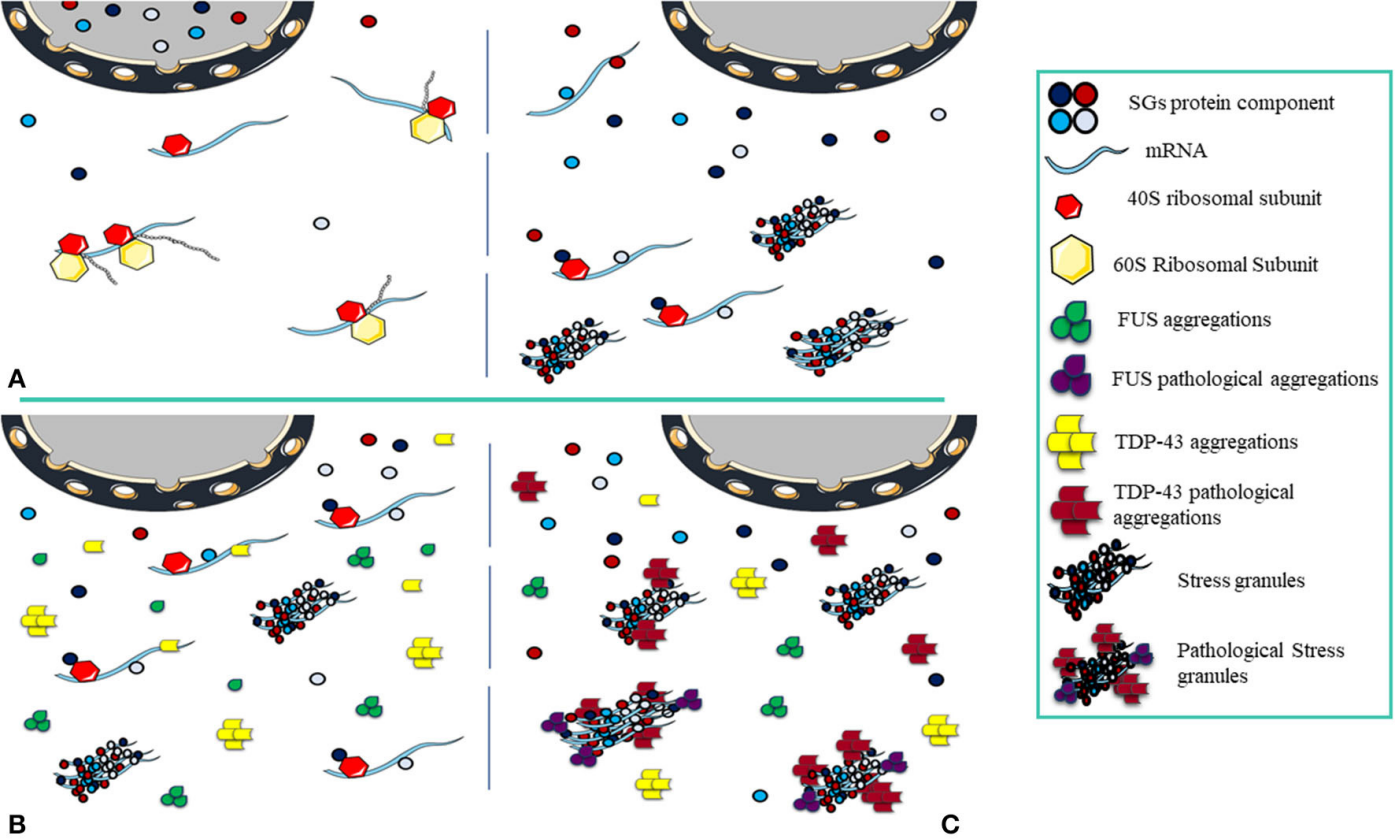
Pathological Stress Granules Formation. **(A)** The cell is in equilibrium, and as stress arrives, nuclear depletion of SGs components occurs, the translation process halts and the structure of transient SGs is formed. Once the stress is relieved, the cell returns to normal and SGs disassemble. **(B)** The cell is exposed to acute stress, the components of SGs and protein aggregations (FUS and TDP-43) localize to the cytoplasm. In this condition, FUS and TDP-43 aggregations have not become pathological and do not interact with SGs, the number of SGs decreases as stress is relieved. **(C)** Under chronic stress, FUS and TDP-43 aggregations become pathological and in interaction with SGs, under the constant presence, pathological effects appear.

The studies were published from 2016 to 2020. In this ward, only ALS patients were sampled, whose numbers were around 50 as their number has not been reported in 5 studies. The average age of the people reported in this section is 47.6 years. Samples used in the studies included human skin fibroblasts in 18 studies, hair follicle cells (Japtok et al., 2015) and human B-lymphoblastoid cells (Daigle et al., 2016) each in one study. The protein component of SGs examined in these studies includes G3BP1, TIA-1, hnRNPA1, TDP-43, PABP-1, FUS, TIAR, eIF4A3, eIF4A1, eIF3, eIF4G, UPF1, UPF3b, HuR, ANXA11, Pur-alpha, which are also summarized in Figure 3. The cell lines used in this study carry missense mutations in SOD1 (Gal et al., 2016; Rajpurohit et al., 2020), TARDBP (Orrù et al., 2016; Loginov et al., 2018; Ratti et al., 2020), FUS (Japtok et al., 2015; Lenzi et al., 2015; Daigle et al., 2016; Ichiyanagi et al., 2016; Lim et al., 2016; Lo Bello et al., 2017; Kamelgarn et al., 2018; Arenas et al., 2020), TDP-43 (Kreiter et al., 2018; Loginov et al., 2018; Feneberg et al., 2020) and ANXA11 (Gieseler et al., 2019) genes and frameshift mutations in FUS (Japtok et al., 2015; Lim et al., 2016) gene and hexanucleotide repeat expansion mutations in C9ORF72 gene (Dafinca et al., 2016; Loginov et al., 2018; Ratti et al., 2020). Studies were conducted in the United States, Italy, Germany, France, Japan, South Korea, India and the United Kingdom.

### SGs: From Assembly to Disequilibrium and Pathogenesis

Inhibition of translation due to stress is the main factor for SGs assembly. The size of the SGs can vary from 100 to 1,000 nm (Kedersha et al., 1999). SG assembly needs inhibition of the assembly of polysomes so that only the 40S ribosome subunit remains attached. Non-translated mRNAs are attached to other RNA binding proteins at the nucleation phase in the SGs formation. These proteins at the nucleation phase, also known as SG nucleators, are generally multi-domain proteins in which the IDR “intrinsically disordered region” and RBD domains are prominent (Kedersha et al., 2005) and facilitate the assembly of SGs (Gilks et al., 2004). The nucleation phase continues with the formation of a repository of mRNPs *in situ*, where it is accompanied by interactions between RNA-RNA and RNA-protein to create a stable form of SG cores. Increasing the interactions with the growth of these cores brings the concentration to the critical level, initiates the phase changes (Kedersha et al., 2005; Molliex et al., 2015; Patel et al., 2015) and creates the primary biphasic core/shell structures. The composition of biphasic SGs creates a larger mature assembly during the microtubule-dependent process (Wheeler et al., 2016). The opposite point of assembling SGs is disassembly. In general, disassembly involves the return of non-translated mRNAs trapped in the SG structures to the translation process and is caused by a variety of factors including chaperones in the stress relief phase (Mazroui et al., 2007), microtubules (Loschi et al., 2009), autophagy mechanisms (Dormann et al., 2010), and post-translational modifications (Stoecklin et al., 2004). Assembling and disassembling of SGs are in equilibrium with polysomes (Kedersha et al., 2000). Since biotic or environmental stress stops translation and increases the number of SGs, relieving stress and increasing the number of translated mRNAs is associated with return to equilibrium and disassembly of SGs (Panas et al., 2016; Marnik and Updike, 2019). When the condition progresses toward disequilibrium, by increasing the assembly of SGs and decreasing the clearance, pathogenic processes would be evolved (Wolozin, 2012; Chen and Liu, 2017) (Figure 3).

This pathogenesis links SGs to a wide range of neurodegenerative diseases including ALS (Colombrita et al., 2009; Volkening et al., 2009; Farg et al., 2013; Manghera et al., 2016; Dreser et al., 2017; Chen and Cohen, 2019), AD (Castellani et al., 2011; Vanderweyde et al., 2012; Ivanov et al., 2016; Maziuk et al., 2018; Silva et al., 2019; Younas et al., 2020), FTD (Hirsch-Reinshagen et al., 2017; Mackenzie et al., 2017; Hock et al., 2018; Montalbano et al., 2020), and FTDP (Vanderweyde et al., 2012). Many of the protein components of SGs such as TIA-1 (Fujita et al., 2008; Volkening et al., 2009; Liu-Yesucevitz et al., 2010; Vanderweyde et al., 2012; Dafinca et al., 2016; Gal et al., 2016; Hirsch-Reinshagen et al., 2017; Mackenzie et al., 2017; Salapa et al., 2018; Chen and Cohen, 2019; Younas et al., 2020), PABP-1 (Fujita et al., 2008; Dormann et al., 2010; Bentmann et al., 2012; McGurk et al., 2014; Dafinca et al., 2016; Gal et al., 2016; Hirsch-Reinshagen et al., 2017; Kamelgarn et al., 2018; Silva et al., 2019), G3BP1 (Vanderweyde et al., 2012; Daigle et al., 2016; Gal et al., 2016; Ichiyanagi et al., 2016; Lim et al., 2016; Manghera et al., 2016; Orrù et al., 2016; Loginov et al., 2018; Mann et al., 2019), eIF4G (Dormann et al., 2010; Lim et al., 2016; Kamelgarn et al., 2018), TTP (Vanderweyde et al., 2012) and TIAR (Colombrita et al., 2009; Lenzi et al., 2015; Lo Bello et al., 2017; Codron et al., 2018; Loginov et al., 2018; Chen and Cohen, 2019; Ratti et al., 2020) have been studied in neurodegenerative diseases. IHC has revealed the colocalization of pathological accumulations of proteins such as TDP-43 (Fujita et al., 2008; Colombrita et al., 2009; Volkening et al., 2009; Liu-Yesucevitz et al., 2010; Bentmann et al., 2012; Vanderweyde et al., 2012; McGurk et al., 2014; Gal et al., 2016; Manghera et al., 2016; Orrù et al., 2016; Chen and Liu, 2017; Hirsch-Reinshagen et al., 2017; Mackenzie et al., 2017; Bennett et al., 2018; Kreiter et al., 2018; Loginov et al., 2018; Chen and Cohen, 2019; Mann et al., 2019; Feneberg et al., 2020; Levin et al., 2020; Montalbano et al., 2020; Ratti et al., 2020; Salapa et al., 2020; Vassileff et al., 2020) or FUS (Farg et al., 2013; McGurk et al., 2014; Japtok et al., 2015; Lenzi et al., 2015; Daigle et al., 2016; Lim et al., 2016; Lo Bello et al., 2017; Hock et al., 2018; Kamelgarn et al., 2018; Arenas et al., 2020) with SGs in neurodegeneration, indicating a strong association of SGs with the pathogenic mechanisms. In this study, by reviewing all studies on SGs and neurodegenerative diseases in humans, we tried to answer the question that SGs can act as nests or sources for these pathological aggregations, or disruption and mutations in the main components of these accumulations can disrupt the balance of SGs.

### ALS and FTD Disorders and SGs

ALS is a neurodegenerative disease specific to motor neurons (MNs), and with progressive loss of upper MNs (UMN) in the motor cortex of the brain and lower MNs (LMN) in the brain stem and the spinal cord (Robberecht and Philips, 2013), muscle weakness and atrophy appear (Hardiman et al., 2017). The main pathological signature of ALS is the presence of inclusion bodies in the cytoplasmic region of MNs. The key component of these inclusions in 95% of cases is TDP-43 in the form of hyperphosphorylated, Ubiquitinated, and truncated (Neumann et al., 2006; Nonaka et al., 2016). FUS and SOD1 are other proteins that can be involved in the formation of these inclusions (Volkening et al., 2009; Farg et al., 2013). TDP-43 and FUS are RNA-binding proteins that are often nuclear localized but can commute between the nucleus and the cytoplasm (Kapeli et al., 2017). The nuclear activity of these proteins is summarized in transcription, pre-mRNA splicing and processing non-coding RNAs (Ederle and Dormann, 2017). In the cytoplasm, these proteins can contribute in the regulation of mRNA stability, mRNA transport, translation, autophagy, and stress response and LLPS (Birsa et al., 2020). SOD1 is a superoxide dismutase enzyme. Mutations in this gene can result in these accumulation (Volkening et al., 2009). Based on bioinformatics studies and overlap in the function of these proteins in pathological aggregations and impairment of mRNA mechanisms, these proteins can be considered as pathological factors in these disorders (Liu-Yesucevitz et al., 2010; Wolozin, 2012; Ramaswami et al., 2013). One of the symptoms of impaired mRNA mechanisms is the disequilibrium of SGs. Numerous studies have been performed on the presence of SGs and their association with pathological aggregations in humans using post mortem tissue (brain, spinal cord) and blood, which are summarized in Table 2. To investigate the presence of SGs in the target tissue, SG markers were used, which include TIA-1, G3BP1, PABP-1, TIAR, HuR, and TTP. Studies of human-derived cell lines have been mostly performed using autopsy skin fibroblasts and differentiation into motor (Dimos et al., 2008). These studies have allowed the study of mutations in the TDP-43, FUS, SOD1, and C9orf72 genes and their effects on cell and other components of pathological aggregations and SGs, which are summarized in Table 3.

**Table 3:** Stress Granules and ALS in human derived cell lines.

	**Year of publication**	**Country**	**Cell line origin**	**Diagnosed neurodegenerative disease (number of patients)**	**Age at biopsy**	**Mutation**	**Mutation type**	**Analysis method(s)**	**Protein component of stress granules**	**Major findings**	**References**
Daigle et al.	2015	United States	Human B-lymphoblastoid cells	ALS	NR	FUS-R521C FUS-R518G	Missense	Culturing human lymphoblastoid cells, Immunofluorescence, Generation of deletion constructs, Western blotting, Quantitative PCR, Assessment of neuronal viability, Propidium iodide staining, TUNEL assay, Stress granule induction, and quantification	Pur-alpha G3BP1 FUS	•Pur-alpha was identified as a new component of stress granules in mutant-carrying cells. •Pur-alpha colocalized along with FUS in structural SGs. •Pur-alpha is essential for the formation of SGs. •FUS mislocalization and toxicity caused by its mutations are suppressed by improper expression of Pur-alpha.	Daigle et al., 2016
Japtok et al.	2015	Germany	Human skin fibroblast hair follicle cells (keratinocyte)	ALS (2 case and 3 control)	ALS Patients with 58 and 29 years old	FUS- R521C FUS- R495QfsX527	Missense frameshift	Generation and expansion of iPSCs, *In vitro* differentiation of embryoid bodies, AP staining and immunofluorescence on iPSC colonies, Immunofluorescence on cortical neurons, Karyotyping, Genotyping, Differentiation of human iPSCs into cortical neurons, Sodium arsenite treatment, Quantification, and statistics	FUS	•Type of FUS mutation determines the quantity of FUS accumulation in stress granules and cellular susceptibility to exogenous stress.	Japtok et al., 2015
Lenzi et al.	2015	Italy	Human skin fibroblast	ALS (3 case and 1 control)	NR	FUS-R514S FUS-R521C FUS-P525L	Missense	Generation and maintenance of human iPSCs, Differentiation of iPSCs into ventral spinal cord neural cells, RT-PCR, RT-qPCR and western blot analyses, Immunostaining and confocal imaging, Quantification of nuclear/cytoplasmic and SG FUS distribution and line scan analysis, TALEN-directed mutagenesis	FUS TIAR	•FUS mislocalization and its application in the structure of stress granules is specific to different types of mutant FUSs and occurs only under stress. •The amount of FUS used in the structure of SGs depends on the type of mutation and the amount of FUS proteins in the cytoplasm.	Lenzi et al., 2015
Gal et al.	2016	United States	Human skin fibroblast	ALS (1 case and 1 control)	ALS Patient with 63 years old Healthy control with 64 years old	SOD1- L144F	Missense	Skin Biopsy and Fibroblast Culture, Fluorescence microscopy, Coimmunoprecipitation assays, Western blotting, *In silico* docking, Stress granule induction and analysis	SOD1 G3BP1 TIA-1 hnRNPA1 TDP-43 PABP-1	•Co-localization was observed between mutant SOD1 (L144F) with G3BP1unlike WT-SOD1 protein in fibroblast cells. •The slight association of mutant SOD1 protein with several other RNA-binding proteins in ALS indicated that these interactions were more specific to the G3BP1 protein. •The RRM domain of G3BP1 and two phenylalanine residues (F380 and F382) are important for desired interactions. •SOD1 mutations and their effects on SG dynamics can be strongly associated with the characteristics and pathogenesis of ALS.	Gal et al., 2016
Dafinca et al.	2016	United kingdom	Human skin fibroblast	ALS(NR)	NR	C9orf72 mutation	HRE	Generation and Culture of iPSC Lines, Assessment of Genome Integrity and Tracking, Sendai Clearance Assay, Pluri Test, Flow Cytometry, Differentiation of iPSCs to MNs, Differentiation of iPSCs to CNs, IHC, Immunoblotting, Propidium Iodide Staining, Mitochondrial Staining, ER Calcium Imaging, RNA-FISH, Electrophysiology, Southern Blotting, qRT-PCR, Repeat-Primed PCR, Electron Microscopy	TIA-1 PABP-1	•Abnormal accumulation of proteins and stress granules was observed in c9orf72 IPSC-derived MNs. •Decreased survival of these cells may be due to disruption of mitochondrial membrane potential and calcium homeostasis, increased ER stress and decreased BCL2 protein levels. •By examining the effects of C9orf72 mutations on calcium signaling pathways, the importance of this pathway as a therapeutic target in neurodegenerative disease was determined.	Dafinca et al., 2016
Lim et al.	2016	South Korea	Human skin fibroblast	ALS (Shaw and Jordan, 1995) Healthy control (Shaw and Jordan, 1995)	ALS Patients ranging from 31 to 57, mean 39 years	FUS-Q519E FUS-G504Wfs FUS-R495	Missense frameshift	Conversion of human skin fibroblasts to iNeurons, Immunocytochemistry and confocal microscopy, Nuclear-cytoplasmic fractionation and immunoblot analysis	FUS eIF4G G3BP1	•Mislocalization of FUS proteins and nuclear clearance were seen in patient-derived cells. •Oxidative stress causes cytoplasmic FUS protein accumulations. •The pathological features of P.Q519E mutation were detected only in patient derived-ineuron cells as opposed to fibroblasts and transfected cells.	Lim et al., 2016
Ichiyanagi et al.	2016	Japan	Human skin fibroblast	ALS (2 case and 1 control)	ALS Patients with 39 and 43 years old	FUS-H517D	Missense	Isolation of Human Skin Fibroblasts and Generation of iPSCs, Motor Neuron Differentiation, Immunocytochemistry, High-Content Analysis, Quantitative RT-PCR, Sequence Analysis, Exon Array for MPCs	FUS G3BP1	•The produced cell line showed several characteristics of neurodegenerative diseases such as FUS mislocalization and SG production against stress. •Aberrant gene expression or incorrect splicing, was detected in MPCs using exon array analysis and CLIP-seq dataset. •The IPSC produced in this study can be well used to assess the characteristics of motor neuron diseases.	Ichiyanagi et al., 2016
Orru et al.	2016	Italy	Human skin fibroblast	ALS (2 case without mutations and 2 mutant carrier and 3 healthy control)	NR	TARDBP-A382T	Missense	Cell culture and treatments, IHC, Transfection, qPCR, Western blot Quantification of cells, forming SGs and SG size, Cell viability assays	TDP-43 HuR G3BP1	•TDP-43 did not participate directly in the structure of SGs, it helps to form SG by regulating the G3BP1 core protein. •A382T mutation meaningfully reduced the number of SGs per cell. •Stress stimulation for cells with the A382T mutation reduced survival due to the loss of TDP-43 function in SGs nucleation. •TDP-43 protein mislocalization was not detected as a pathological factor in cell death.	Orrù et al., 2016
Lo Bello et al.	2017	Italy	Human skin fibroblast	ALS (2 case without mutations and 2 mutant carrier and 2 healthy control)	NR	FUS-P525L	Missense	Skin Biopsy and Fibroblast Culture, Stress Treatment, Subcellular Fractionation, SDS-PAGE and Western Blotting, Immunofluorescence, Subcellular FUS Expression, Time Course of the Number of Fibroblasts Containing Stress Granules, Evaluation of the Number of Stress Granules per Cell	FUS TIAR	•High nuclear FUS expression was observed in fibroblasts in both controls and patients. Protein placement in mutant carriers was seen in both nucleus and cytoplasm (mostly cytoplasm). •Stress treatment caused the mis-localization of large amounts of FUS proteins in the cytoplasm and placement in the structure of SGs, instead time-dependent reduction was seen in all cases. •Fibroblasts mutant carriers had more SGs than ALS and control samples and persevere in the cell longer.	Lo Bello et al., 2017
Codron et al.	2018	France	Human skin fibroblast	ALS (6 case and 4 control)	ALS Patients ranging from 54 to 76, mean 63.8 years Healthy controls with a mean age of 59.7	NR	NR	Cell culture, Cell growth assay, Immunofixation, 3D fluorescence microscopy, Super resolution microscopy (dStorm), Reactive oxygen species detection Reactive oxygen species detection	TDP-43 TIAR	•There was no difference between fibroblast cell growth, shape and spreading in patients and controls cells. •Mislocalization and accumulation of TDP-43 were not detected in patients and controls cells. •The cytoskeleton appeared completely normal in cells and no difference was observed in the distribution of mitochondria. •The rate of ROS production and its stress response in patient-derived cells and controls showed similarity. •Patient-derived fibroblasts are not suitable for pathological and prognostic studies of ALS.	Codron et al., 2018
Kamelgarn et al.	2018	United States	Human skin fibroblast	ALS (6 case and 5 control)	ALS Patients ranging from 26 to 58, mean 42.1 years	FUS-R521G FUS-P525R	Missense	Skin Biopsy and Fibroblast Culture, Protein Translation Assays, NMD Activity Assays	FUS eIF4A3 eIF4A1 eIF3 eIF4G UPF1 UPF3b PABP-1	•Development of a new protocol for the separation of positive cytoplasmic granules. •The mutant FUS in fibroblast cell line derived from ALS's and can interfere with the translation process. •The mutant FUS interfered with the automatic regulation of NMD and increased its activity by increasing its promoting factors (UPF1, UPF3b) and decreasing its negative regulatory factors (UPF3a).	Kamelgarn et al., 2018
Kreiter et al.	2018	Germany	Human skin fibroblast	ALS (2 case and 4 control)	NR	TDP-43-S393L TDP-43-G294V	Missense	Generation of iPSC lines, Trilineage differentiation potential, Karyotyping, Genotyping Differentiation of human NPCs to spinal motor neurons, Immunofluorescence of spinal motor neurons, Microfluidic chambers, Live cell imaging, Tracking analysis, Static analysis of cell organelles, Electrophysiology	TDP-43	•In the early stages of neuronal differentiation, no difference was seen between TDP-43 mutant cell lines and WTs. •Neuronal loss and pathological neurofilament abnormalities were seen in the aging stage in mutant TDP-43 cell lines. •Abnormal phenotypes in terms of shape, size, and motility were observed in mitochondria and lysosomes that were not due to mis-localization or accumulation of TDP-43 in the motor neurons carrying the TDP-43 mutations in the aging phase. •Axon trafficking in motor neurons was improved by D-sorbitol, but no TDP-43 accumulations or mis-localizations were observed. •S393L and G294V Mutations can cause motor neuron degeneration but are independent of the cytoplasmic accumulation of TDP-43.	Kreiter et al., 2018
Colombrita et al.	2018	Italy	Human skin fibroblast	ALS (6 case and 3 control)	NR	TARDBP -A382T C9orf72 mutation	Missense HRE	Cell culture and treatments, Quantification of cells forming SGs and SG size	TDP-43 TIAR	•Stress granules formed by arsenite treatment are larger than SGs formed by acute stress. •SGs examined in C9orf72 and TARDBP mutant cells were different in size and number. •Arsenic-induced SGs used TDP-43 in mutant cells, while TDP-43 was not found in acute stress SGs. •Filamentous and round pTDP-43 inclusions were found in mutant-carrying cells after chronic arsenite stress.	Theme 4 Human cell biology and pathology, 2018
Dafinca et al.	2018	United kingdom	Human skin fibroblast	ALS (2 case and 2 control)	NR	TDP-43M337V TDP-43 I383T	Missense	Skin Biopsy and Fibroblast Culture, Generation of iPSC lines, HI-FI CRISPR/Cas9, Stress granule analysis, Nucleo-cytoplasmic Transport investigation	TDP-43 G3BP1	•TDP-43 mutations devastate protein degradation in IPSC derived MNs. •TDP-43 mutations have a devastating effect on the mechanism of protein transport between the nucleus and the cytoplasm.	Theme 4 Human cell biology and pathology, 2018
Hedges	2019	United Kingdom	Human skin fibroblast	ALS(NR)	NR	ANXA11- D40G ANXA11- G38R ANXA11- R235Q	Missense	Opera Phenix imaging platform, super resolution microscopy, live imaging	TDP-43 ANXA11	•TDP-43 protein mislocalization was detected in ANXA11 mutant motor neurons. •Altering the location of ANXA11 protein with stress granules (reduction in co-localization).	Gieseler et al., 2019
Rajpurohit et al.	2020	India	Human skin fibroblast	ALS(NR)	NR	SOD1-L39R	Missense	Reprogramming of iPSCs and Culture, Differentiation of iPSCs into Astrocytes and Motor Neurons, Stress Granule Dynamics, Endoplasmic Reticulum Stress, Autophagy Studies, Non-Cell Autonomous Neurotoxicity Studies, IHC Analysis,	SOD1 G3BP1	High expression of G3BP1 and co-localization with SOD1-L39R in ALS's MNs and astrocytes is associated with increased AIF1-mediated autophagy activity and caspase 7/3 upregulation.	Rajpurohit et al., 2020
Arenas et al.	2020	United States	Human skin fibroblast	ALS (5 case and 5 control)	ALS Patients ranging from 26 to 58, mean 39.4 years.	FUS-R521G FUS- P525R	Missense	Patient skin fibroblast isolation and culture, Generation of the anti-acetylated-K510 FUS antibody	FUS	•FUS acetylations in lysine 510, located in the NLS sequence, disrupts the interaction between FUS and Transportin-1 and results in FUS mis-localization in the cytoplasm resembling SG aggregations. •ALS fibroblasts showed greater acetylations in lysine compared to controls.	Arenas et al., 2020
Feneberg et al.	2020	United Kingdom	Human skin fibroblast	ALS (1)	NR	TDP-43M337V	Missense	Human cellular models, Mass spectrometry and bioinformatics, IHC and microscopy, Stress granule analysis, Immunoprecipitation and immunoblotting, Ultrafiltration liquid chromatography and extracellular vesicle Characterization, Transmission electron microscopy	TDP-43 eIF4A1	TDP-43M337V can increase the formation of stress granules by degrading interprotein interactions to increase binding to eIF4A1 and endoplasmic reticulum chaperone Grp78.	Feneberg et al., 2020
Ratti et al.	2020	Italy	Human skin fibroblast	ALS (2 case and 1 control)	ALS Patients with 56 and 48 years old. Healthy control with 45 years old.	TARDBP -A382T C9orf72 mutation	Missense HRE	iPSC-derived motoneurons, Arsenite treatment, Cell viability assay, Immunofluorescence, Quantitative analyses of SG, Colocalization image analysis, TEM, Quantitative analysis of TEM data	TDP-43 TIAR	•Recruitment of TDP-43 as pTDP-43 in SGs structure due to chronic stress and increase in P62. •Genetics play a key role in responding to cellular stress and the size and number of SGs. •Disruption of autophagy mechanisms may be involved in TDP-43 aggregations. •Arsenite stress relief was associated with a decrease in number of SGs and TDP-43 aggregations in 72 h, but P62 remained, so Disruption of autophagy mechanisms may be involved in TDP-43 aggregations.	Ratti et al., 2020

*SG, Stress Granule; ALS, Amyotrophic lateral sclerosis; PCR, polymerase chain reaction; iPSC, Induced pluripotent stem cells; RT-PCR, Reverse Transcriptase PCR; Q-PCR, Quantitative PCR; TALEN, Transcription activator-like effector nucleases; HRE, hexanucleotide repeat expansion; CN, cortical neurons; MN, Motor Neurons; IHC, Immunohistochemistry; ER, Endoplasmic Reticulum; qRT-PCR, Quantitative Reverse Transcription PCR; MPC, motor neuron precursor cell; NMD, Non-sense Mediated mRNA Decay; NPC, neural precursor cell; HI-FI, high-fidelity; NLS, nuclear localizing sequence; TEM, Transmission Electron Microscopy*.

TAR DNA-binding protein 43 (TDP-43), product of the TARDBP gene, plays the largest role in the formation of pathological aggregations. Most of the changes observed in this protein in aggregations include phosphorylation (Liu-Yesucevitz et al., 2010; Bentmann et al., 2012; Cohen et al., 2015; Hirsch-Reinshagen et al., 2017; Ratti et al., 2020), acetylation (Cohen et al., 2015), and cleavage at N/C terminals (Bentmann et al., 2012; Cohen et al., 2015). General observations suggest that hyperphosphorylation and acetylation may predispose TDP-43 to accumulation. TDP-43 has been studied in both full-length and cleaved (Cohen et al., 2015) forms in aggregations in spinal cord (McGurk et al., 2014; Chen and Cohen, 2019) and brain (Liu-Yesucevitz et al., 2010; Manghera et al., 2016; Hirsch-Reinshagen et al., 2017; Vassileff et al., 2020). In studies of colocalization of TDP-43 with SGs, it is most often colocalized with TIA-1, PABP-1, G3BP1, TIAR, and HuR, which are markers for the presence of SGs in cell and are the core proteins in nucleation phase in the assembly of SGs (Kedersha et al., 2005). In addition, in MNs differentiated from fibroblasts, the effect of TDP43 on nucleation phase and interaction with G3BP1 in CORE formation has been determined (Orrù et al., 2016). TDP-43 is colocalized with STAUFEN and FMRP, which play important roles in mRNA mechanisms. STAUFEN (Volkening et al., 2009; Vassileff et al., 2020), encoded by the STAU1 gene, is involved in the transport of mRNA to different subcellular compartments and organelles (Thomas et al., 2005). FMRP is involved in the nucleocytoplasmic shuttling and dendritic localization of mRNA (Antar et al., 2005). So far, the effects of TDP-43 mutants including S393L (Kreiter et al., 2018), G294V (Kreiter et al., 2018), M337V (Loginov et al., 2018; Feneberg et al., 2020), I383T (Loginov et al., 2018) and TARDBP-A382T (Orrù et al., 2016; Loginov et al., 2018; Ratti et al., 2020) mutations in human-derived cell line studies have been considered. S393L, G294V missense mutations can cause neurodegeneration in MNs in a TDP-43 accumulation independent manner (Kreiter et al., 2018). The TDP-43 M337V mutant can increase the assembly of SGs by interfering with the function of eIF4A1 and endoplasmic reticulum chaperone Grp78 (Feneberg et al., 2020). In contrast, TARDBP-A382T mutation due to loss of TDP-43 function significantly has reduced the number of SGs in cells (Orrù et al., 2016).

FUS (fused in sarcoma) is present in pathological aggregations with a lower percentage than TDP-43 and its association and effects on SGs have been studied. The mechanism of FUS toxicity is not fully understood, but due to its cytoplasmic localization, loss of nuclear activity and acquisition of cytoplasmic function might be involved (Kino et al., 2015). Studies in post-mortem tissue have shown the association of FUS aggregations with SGs through colocalization with ATAXIN2 (Farg et al., 2013) and PABP-1 (McGurk et al., 2014). FUS aggregations are directly affected by the type of mutation, benign and malignant (Japtok et al., 2015). The amount of mislocalization and recruitment in the structure of SGs is related to the type of mutation (Lenzi et al., 2015). FUS in the cell carrying the P525L mutation has more cytoplasmic localization than in control, and when exposed to stress, this localization increases leading to nuclear depletion (Lim et al., 2016). After stress relief, the mutant carrier cell needs more time to return to normal than control. SGs are more numerous in this cell and have a longer persistence, which indicates the direct effect of FUS mutations on SGs (Lo Bello et al., 2017). Post-transcription modifications, such as acetylation in lysine 510, which is located in the NLS sequence, disrupts the interaction between FUS and transportin1, causing its cytoplasmic mislocalization, which is more common in the pathogenesis of ALS than in controls (Arenas et al., 2020). The localization of SOD1 has been confirmed in both mutant and WT form with TDP-43 accumulations in spinal cord motor neurons (Volkening et al., 2009). Mutations that occur in SOD1 can also affect the dynamics of SGs. The mutant types SOD1-L144F (Gal et al., 2016) and SOD1-L39R (Rajpurohit et al., 2020) colocalize with G3BP1 in fibroblast-derived motor neurons, whereas SOD1-WT does not colocalize with SGs (Gal et al., 2016; Rajpurohit et al., 2020).

Hexanucleotide repeat expansion (HRE) is another common mutation in ALS that is associated with an increase in the number of G4C2 repeats in the C9orf72 gene. The number of repeats in normal people are between 20 and 30, but in people with mutations, the number of repeats increases to hundreds (Khan et al., 2012). Three mechanisms explain the effect of C9orf72 mutations on SGs.

1. C9orf72-related RNA transcripts accumulate in the nucleus and cytoplasm causing sequestration of RNA-binding proteins, including proteins involved in SG dynamics (Rossi et al., 2015; Dafinca et al., 2016). Colocalization of PABP-1 with TDP-43 inclusions was higher in ALS patients with C9orf72 mutation (67%) than in patients with ATXN2 mutation (47%) and patients without any mutation (36%) (McGurk et al., 2014).
2. The effect of mutations on C9orf72 protein function and the destruction of interactions with other proteins is another proposed mechanism. DENNL72 is another name for C9orf72, which stands for differentially expressed in normal and neoplastic cells and One of the molecular roles envisaged for it is acting as a guanine nucleotide exchange factor (GEF) (Levine et al., 2013). The interaction of C9orf72 as GEF with Rab proteins (Tang, 2016) is involved in autophagy and cellular trafficking mechanisms such as Rab7 and Rab11 (Farg et al., 2014) and disruption of these pathways can lead to cytoplasmic accumulations of TDP-43 (Ratti et al., 2020) and decreased clearance of SGs (Monahan et al., 2016).
3. The transcript of the mutant C9orf72 gene containing GGGGCC repeats can be translated during the non-ATG translation mechanism and produce 5 different types of dipeptide repeats (DPRs) (Mori et al., 2013). Among these, arginine-containing dipeptide repeats can interact with a number of SG protein components that have the IDR domain. DPRs containing glycine and proline also play a role in the assembly of SGs by inhibiting the translation and phosphorylation of eIF2a and G3BP1 (Lee et al., 2016).

FTD is one of the most common types of dementia that affects people under the age of 65 (Bird et al., 1999) and represents a diverse range of subtypes with neurodegenerative disorders such as FTLD (Faber, 1999). Due to common characteristics in clinical observations, ALS and FTD now form a broad continuum of neurodegeneration that can occur in an individual or a family. Like ALS, studies on post-mortem tissue, specifically the brain of FTD patients, have revealed the association and development of the disease with SGs (Dormann et al., 2010; Liu-Yesucevitz et al., 2010; Hirsch-Reinshagen et al., 2017; Mackenzie et al., 2017). Most pathological mechanisms and aggregations such as TDP43, FUS, and C9orf72 mutations overlap with ALS. But FTD has some distinctive features. No colocalization was observed between PABP-1 and eIF4G with cytoplasmic neuronal inclusions in FTLD-TDP brain (Dormann et al., 2010), and acetylated and full-length pTDP-43 have no effects in the pathogenesis of FTLD-TDP (Cohen et al., 2015).

### AD and SGs

AD is a chronic neurodegenerative disease that is accompanied by death of neurons and loss of synapses in the cerebral cortex and certain subcortical regions of the brain (Tiraboschi et al., 2004; Burns and Iliffe, 2009). The most common form of dementia is AD, which occurs with abnormal structures (Wang et al., 2018), extracellular senile plaques, composed mainly of small proteins called Aβ42 (Bate et al., 2006), and intraneuronal neurofibrillary tangles, which are the result of accumulations of hyperphosphorylated Tau proteins (Bancher et al., 1989). Tau proteins are a group of six protein isoforms produced by alternative splicing of the MAPT (microtubule-associated protein Tau) gene (Goedert et al., 1989). The main function of Tau proteins is to maintain the stability of microtubules in axons due to their high expression in CNS neurons (Haritani et al., 1994), which are highly soluble in the cytoplasm and contribute to the dynamic and functions of microtubules (Arendt et al., 2016). Tau undergoes many post-translational modifications, including hyperphosphorylation (Grundke-Iqbal et al., 1986), acetylation (Min et al., 2010), C-terminal truncation (Zhao et al., 2016), and n-glycosylation (Wang et al., 1996), which can play a role in regulating its localization and functions. Usually, Tau is non-phosphorylated in interaction with microtubules in the axons. When stress is applied, it is phosphorylated near the microtubule binding domain and loses its ability to bind to microtubules (Trinczek et al., 1995). The hyperphosphorylated form of Tau is seen in all six isoforms in neurofibrillary tangles (Hernández and Avila, 2007). Hyperphosphorylation increases the affinity of Tau proteins for each other and binds together to form oligomers and misfolded Taus. Oligomers bind to other units to form Tau deposits, which are the Elements of NFTs that bind together to form NFTs (Shafiei et al., 2017).

Tau can act as a negative regulator of protein translation by binding to ribosomes and reducing protein synthesis (Meier et al., 2016). Stopping translation and providing RNA-binding proteins to mRNA are key elements in the formation of SGs. TIA-1 and TTP are among the core nucleation proteins. The size of Tau aggregations is directly related to TIA-1 colocalization. The larger the Tau aggregations, the greater the rate of TIA-1 colocalization (Vanderweyde et al., 2012). PABP-1 and DDX6 are also proteins found near Tau aggregations in the temporal cortex neurons of AD (Silva et al., 2019). The association of aggregations between RNA-binding proteins and the formation of SGs with Tau aggregates has not been well-studied. Tau aggregations might promote the dynamic equilibrium of SGs toward further assembly or to SGs disequilibrium pave the way for the accumulation of Tau proteins. The formation of RBPs pathological aggregations close to the Tau pathological aggregates supports this hypothesis (Maziuk et al., 2018). Tau proteins have interaction with rps6, which is a component of the 40s ribosome complex, and affects translation inhibition (Koren et al., 2019). Notably, hippocampal neurons in AD patients have more positive rps6 granules than controls (Castellani et al., 2011). Moreover, the colocalization of rps6 with TIA-1 and PABP-1 in basophilic inclusions may indicate one of the main shared mechanisms between Tau aggregations and stress granules (Fujita et al., 2008). The association of TDP-43 oligomers with Tau aggregations has also been suggested as one of the interactions involved in the pathogenesis of Tau (Montalbano et al., 2020).

### MS and SGs

MS is a demyelinating disease in which the cover of nerve cells in the brain and spinal cord is damaged (Noyes and Weinstock-Guttman, 2013). The causative mechanism of the disease is summarized in the destruction of myelin sheath by the immune system and defects in myelin-producing cells (Nakahara et al., 2011). The disease has three main features, including the formation of lesions in the CNS, inflammation and destruction of the myelin sheath (Compston and Coles, 2008). Neurodegeneration is also an important feature in the pathology of MS (Frohman et al., 2006; Lassmann and van Horssen, 2011), but so far, no specific mechanism has been proposed for it. The impact of SGs on neurodegeneration and neurodegenerative diseases has been fully discussed so far. Heterogeneous nuclear ribonucleoprotein A1 (hnRNPA1) is a major component in the formation of SGs (Guil et al., 2006) and is discussed as a factor in the pathogenesis of autoimmune mediated CNS neurological diseases and as a link between SGs and autoimmune neurological diseases (Douglas et al., 2016). The interaction between TIA-1 and hnRNPA1 in cytoplasmic granules and its nuclear depletion in MS patients is significant. The aggregations of TIA-1 in the structure of large SGs in the cytoplasm can be a link between degeneration in neurons and MS (Salapa et al., 2018). Nuclear depletion of TDP-43 and interaction with hnRNPA1 and colocalization of both in the structure of SGs also emphasizes the importance of SGs in MS (Levin et al., 2020; Salapa et al., 2020).

### Bioinformatics Perspective

**Figure 4:**
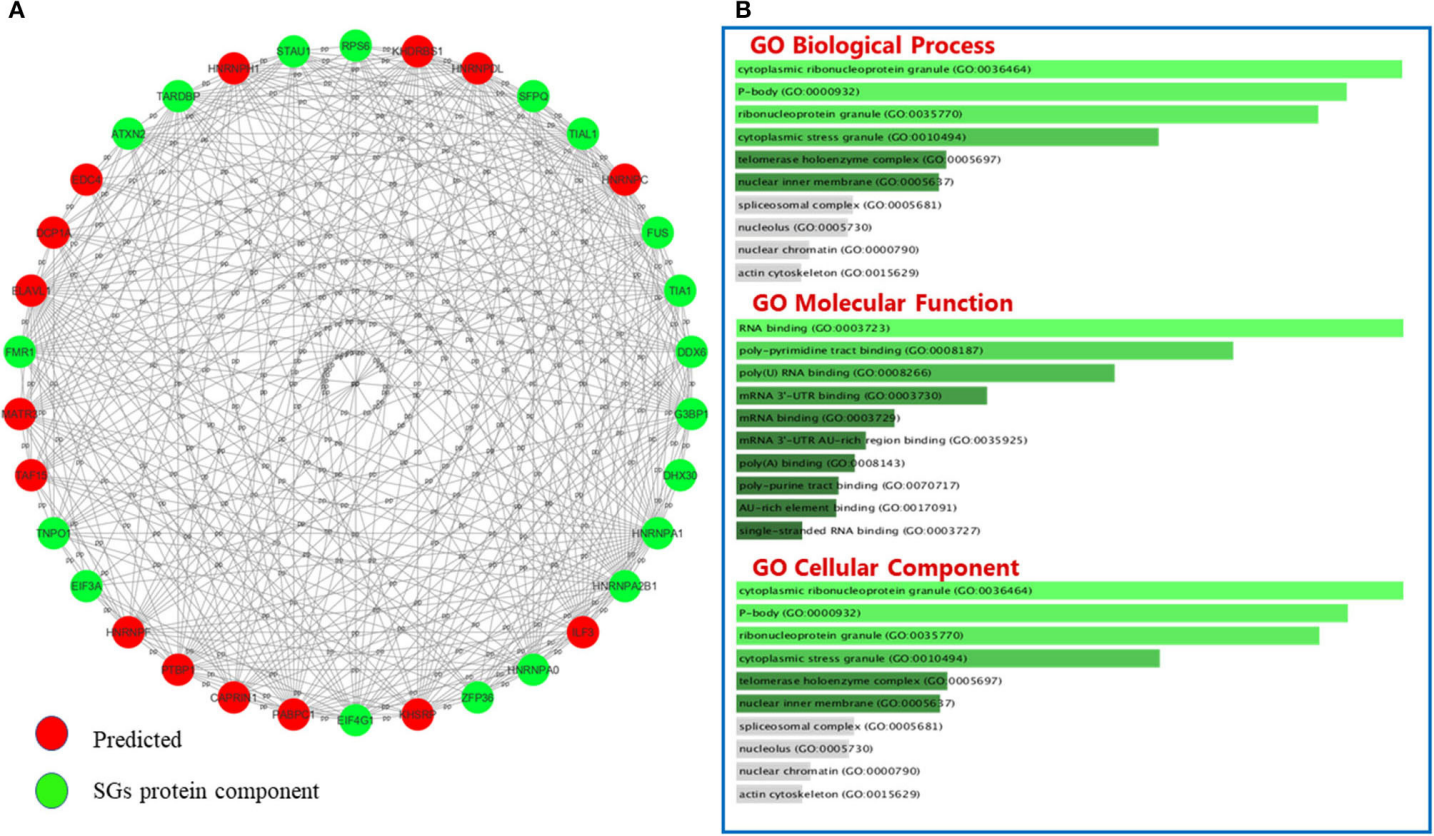
The interactions between SGs protein components and predicted proteins and GO analysis on predicted ones in neurodegenerative disease. **(A)** Fifteen new proteins were predicted by interaction with SGs protein components using the Cytoscape string-db plugin on data extracted from articles. **(B)** Fifteen new proteins were predicted by interaction with SGs protein components using the Cytoscape string-db plugin on data extracted from articles. GO analysis was performed on the predicted proteins in three biological processes: molecular function and cellular component. The length of each bar indicates the importance in that particular category sorted by *p*-value. Note that the lower the color intensity of the bars, the greater the relationship with that category.

GO classifies relationships between genes by annotating and categorizing them into three levels: biological process, molecular function, and cellular component. Biological process describes the cellular or physiological role performed on a larger scale by a gene in relation to other genes. The molecular function describes the molecular activity of the desired gene, and the cellular component determines where the gene product executes its function (The Gene Ontology Consortium., 2019). GO analysis was performed on the list of genes associated with SGs in neurodegenerative diseases (Figure 3A) and the list of genes associated with SGs in ALS disease was extracted from human derived cell lines studies (Figure 3C) using Enrichr's web-based tools and services (Kuleshov et al., 2016). Most of the proteins in the structure of SGs are RBPs (Kedersha et al., 2005), and GO analysis confirms this. According to the GO biological process, the connections that can be made in one biological pathway with other proteins are more involved in the RNA metabolic pathways and, as expected, all of these proteins act in conjunction with RNAs, including cytoplasmic mRNA, body assembly, and RNA splicing. Further use of these proteins in the structure of ribonucleoprotein granules, cytoplasmic SGs and cytoplasmic ribonucleoprotein granules was confirmed by GO cellular component analysis. In ALS, the biological association of these proteins with other proteins, based on the GO biological process, significantly confirmed their role in RNA export from nucleus. This was expected, given that these proteins are mostly shuttling between nucleus and cytoplasm. Gene prediction was performed on the gene list extracted in neurodegenerative disease in Table 3 by string-db cytoscape plugin (Doncheva et al., 2019). GO biological process showed these proteins interact with other proteins in p-bodies, cytoplasmic ribonucleoprotein granules and cytoplasmic SGs. Overall, bioinformatics analysis determined the association of these proteins and genes with RNA-related mechanisms that are specifically involved in the formation and assembly of ribonucleoprotein granules. Their functional position is also shared between nucleus and cytoplasm and is an evidence to trafficking and shuttling of SGs protein components between them (Figure 4).

## Conclusions

Equilibrium is the most important point in SGs. SGs are in the nature of the cell and are considered as cell solutions to stress. They are temporary constructions and when the stress is relieved, they are disassembled and reduced in number, and the cell condition returns to normal. If under any circumstances the presence of SGs becomes permanent and leads to disequilibrium, in interaction with pathological aggregations such as TDP and FUS aggregations, they can lead to pathological conditions such as neuron degeneration. SGs have been studied in many neurodegenerative diseases in humans, including ALS, FTD, AD, and MS. These studies have indicated common features in SG biology among neurodegenerative diseases. At least 15 proteins have been predicted to interact with the protein components of SGs in mentioned neurodegenerative disorders. Therefore, it seems that SG biology is common between these disorders. Yet, some components of SGs might be specific to these disorders. Based on the rarity of comparative analysis between these disorders, it is not possible to male conclusive interpretations in this regard. We have tried to provide a comprehensive summary of these studies and an overview of SGs in neurodegenerative diseases. To conclude, more studies can be done in diseases such as AD and MS and the association of the Tau protein with other protein components of SGs or the association of inflammatory pathways with the formation of SGs could also be assessed.

## Author Contributions

MT, SG-F, and MR wrote the manuscript and contributed in study design. MA, MS, HS, and AJ contributed in the data collection, designed the tables and figures. All authors approved the manuscript.

## Conflict of Interest

The authors declare that the research was conducted in the absence of any commercial or financial relationships that could be construed as a potential conflict of interest.

## Abbreviations

SGs: Stress granules
ALS: amyotrophic lateral sclerosis
FTD: frontotemporal degeneration
AD: Alzheimer's diseases
MS: multiple sclerosis
PRISMA-ScR: Preferred Reporting Items for Systematic Reviews and Meta-Analyses Extension for Scoping Reviews
MeSH: medical subject heading
GO: Gene ontology
FTLD: frontotemporal lobar degeneration
NIFID: neuronal intermediate filament inclusion disease
BIBD: basophilic inclusion body disease
MND: motor neuron disease
IHC: immunohistochemistry
IP: immunoprecipitation
MNs: motor neurons
LMN: lower MNs
TDP-43: TAR DNA-binding protein 43
HRE: Hexanucleotide repeat expansion
GEF: guanine nucleotide exchange factor
hnRNPA1: Heterogeneous nuclear ribonucleoprotein A1.
