# Supplementary material for: Stress Granules and Neurodegenerative Disorders: A Scoping Review
Source: Front Aging Neurosci. 2021 Jun 23;13:650740. doi: 10.3389/fnagi.2021.650740 (PMC8261063; doi:10.3389/fnagi.2021.650740)
Supplement: Supplementary file 1 [file Table_1.docx]

| Database | Search keywords |
| --- | --- |
| Pubmed | ("lncRNA"[Title] OR "lncRNA"[Title/Abstract] OR ("lincRNA"[Title] OR "lincRNA"[Title/Abstract]) OR ("lincRNAs"[Title] OR "lincRNAs"[Title/Abstract]) OR ("long non-coding RNA"[Title] OR "long non-coding RNA"[Title/Abstract]) OR "long untranslated RNA"[Title/Abstract] OR ("long intergenic non-coding RNA"[Title/Abstract] OR "long intergenic non-coding RNA"[Title]) OR ("long intergenic noncoding RNA"[Title/Abstract] OR "long intergenic noncoding RNA"[Title]) OR ("long noncoding RNA"[Title] OR "long noncoding RNA"[Title/Abstract]) OR ("long intergenic non-protein coding RNA"[Title] OR "long intergenic non-protein coding RNA"[Title/Abstract]) OR ("long non-protein coding RNA"[Title] OR "long non-protein coding RNA"[Title/Abstract]) OR "rna, long noncoding"[MeSH Terms]) AND ("alzheimer*"[Title] OR "alzheimer*"[Title/Abstract] OR "Dementia"[Title] OR "Dementia"[Title/Abstract] OR "Cognitive"[Title] OR "Cognitive"[Title/Abstract] OR "Cognition"[Title] OR "Cognition"[Title/Abstract] OR "Memory"[Title] OR "Memory"[Title/Abstract] OR "senil*"[Title] OR "senil*"[Title/Abstract] OR ("Alzheimer Disease"[MeSH Terms] OR "Dementia"[MeSH Terms] OR "Cognitive Dysfunction"[MeSH Terms] OR "Memory Disorders"[MeSH Terms])) |
| Embase | Session Results  .......................................................  No. Query Results Results Date  #29. #15 AND #28 611 10 Jan 2021  #28. #16 OR #17 OR #18 OR #19 OR #20 OR #21 OR #22 OR 32,941 10 Jan 2021  #23 OR #24 OR #25 OR #26 OR #27  #27. 'long untranslated rna'/exp 30,430 10 Jan 2021  #26. 'long non-protein coding rna':ti,ab 18 10 Jan 2021  #25. 'long intergenic non-protein coding rna':ti,ab 194 10 Jan 2021  #24. 'long intergenic non-protein coding rna':ti,ab 194 10 Jan 2021  #23. 'long noncoding rna':ti,ab 6,359 10 Jan 2021  #22. 'long intergenic noncoding rna':ti,ab 204 10 Jan 2021  #21. 'long intergenic non-coding rna':ti,ab 248 10 Jan 2021  #20. 'long untranslated rna':ti,ab 2 10 Jan 2021  #19. 'long non-coding rna':ti,ab 8,457 10 Jan 2021  #18. 'lincrnas':ti,ab 683 10 Jan 2021  #17. 'lincrna':ti,ab 902 10 Jan 2021  #16. 'lncrna':ti,ab 19,679 10 Jan 2021  #15. #1 OR #2 OR #3 OR #4 OR #5 OR #6 OR #7 OR #8 OR 1,106,156 10 Jan 2021  #9 OR #10 OR #11 OR #12 OR #13 OR #14  #14. 'memory disorder'/exp 80,999 10 Jan 2021  #13. 'senil*':ti,ab 22,943 10 Jan 2021  #12. 'memory':ti,ab 331,541 10 Jan 2021  #11. 'cognition':ab 91,632 10 Jan 2021  #10. 'cognition':ti 20,105 10 Jan 2021  #9. 'mild cognitive impairment'/exp 28,283 10 Jan 2021  #8. 'cognitive':ab 494,583 10 Jan 2021  #7. 'cognitive':ti 143,889 10 Jan 2021  #6. 'dementia'/exp 374,466 10 Jan 2021  #5. 'dementia':ab 145,861 10 Jan 2021  #4. 'dementia':ti 67,641 10 Jan 2021  #3. 'alzheimer disease'/exp 206,312 10 Jan 2021  #2. 'alzheimer*':ab 188,823 10 Jan 2021  #1. 'alzheimer*':ti 99,698 10 Jan 2021  ....................................................... |
